# Multiple Gastric Gastrointestinal Stromal Tumors in a Patient with Neurofibromatosis Type 1

**DOI:** 10.1155/2016/1515202

**Published:** 2016-06-07

**Authors:** Makoto Tomatsu, Jun Isogaki, Takahiro Watanabe, Kiyoshige Yajima, Takuya Okumura, Kimihiro Yamashita, Kenji Suzuki, Akihiro Kawabe, Akira Komiyama, Seiichi Hirota

**Affiliations:** ^1^Department of Surgery, Fujinomiya City General Hospital, 3-1 Nishiki-cho, Fujinomiya, Shizuoka 418-0076, Japan; ^2^Department of Diagnostic Pathology, Fujinomiya City General Hospital, 3-1 Nishiki-cho, Fujinomiya, Shizuoka 418-0076, Japan; ^3^Department of Surgical Pathology, Hyogo College of Medicine, 1-1 Mukogawa-cho, Nishinomiya, Hyogo 663-8501, Japan

## Abstract

Gastrointestinal stromal tumors (GISTs) are relatively common in neurofibromatosis type 1 (NF 1) patients. Approximately 90% of GISTs associated with NF 1 are located in the small intestine, while sporadic GISTs are most commonly located in the stomach. Here we report an extremely rare case of an NF 1 patient with multiple gastric GITs (90 or more) but without multiple small intestinal tumors. A 63-year-old female patient who had a history of NF 1 underwent surgery for a gastric neuroendocrine tumor and gastric submucosal tumor (SMT). During the operation, multiple small nodules were identified on the serosal surface of the upper stomach. SMT and multiple nodules on the serosal surface were diagnosed as GISTs consisting of spindle cells positive for KIT, CD34, and DOG-1. Both GIST and the normal gastric mucosa showed no mutations not only in the c-*kit* gene (exons 8, 9, 11, 13, and 17) but also in the* PDGFRA* gene (exons 12, 14, and 18). This patient is being followed up without the administration of a tyrosine kinase inhibitor.

## 1. Introduction

Neurofibromatosis type 1 (NF 1) is one of the most common autosomal dominant traits, with a rate of occurrence of approximately 1 in 4000 in the general population [1]. The cause of NF 1 is a mutation in the* NF 1* gene that encodes neurofibromin. Because neurofibromin inhibits Ras oncogene activity, the loss of neurofibromin function results in Ras activation and subsequent tumor formation [2].

Gastrointestinal stromal tumors (GISTs) are relatively common with prevalences estimated to vary from 5% to 30% in NF 1 patients [1]. Approximately 90% of GISTs associated with NF 1 are located in the small intestine, and only 5.4% are located in the stomach [3].

In this paper, an extremely rare case of multiple gastric GISTs in an NF 1 patient is reported.

## 2. Case Report

**Figure 1:**
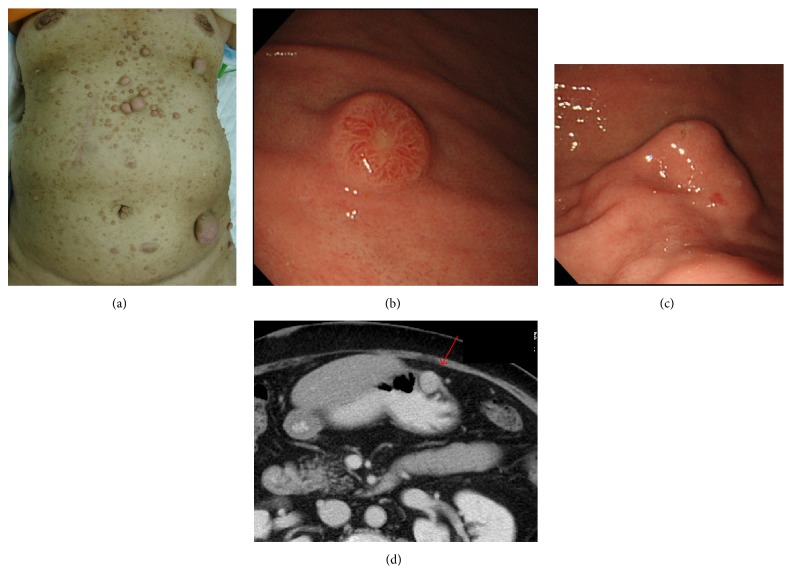
(a) There are multiple neurofibromas and some cafe-au-lait spots all over the body skin. (b) Upper gastrointestinal endoscopy shows neuroendocrine tumor located on posterior side of upper gastric wall. The size was about 10 mm. (c) And submucosal tumor located on greater curvature side of middle gastric wall. The size was about 30 mm. (d) Computed tomography showed only a SMT (arrow); the size was about 30 mm and the surface was smooth. Other lesions could not be pointed out.

A 63-year-old female was examined for dysphagia. She and her father had a history of NF 1. The patient presented with multiple neurofibromas and some cafe-au-lait spots all over her body (Figure 1(a)).

Upper gastrointestinal endoscopy revealed a neuroendocrine tumor (NET) located on the posterior side of the upper gastric wall and a submucosal tumor (SMT) located on the greater curvature of the middle gastric wall (Figures 1(b) and 1(c)). Computed tomography (CT) indicated only SMT, which was approximately 30 mm in diameter and had a smooth surface (Figure 1(d)). CT did not show NET or any other lesion.

Preoperative diagnosis was a gastric NET in combination with a gastric SMT suspected to be GIST. Laparoscopic proximal gastrectomy with D1+ lymph node dissection for NET and partial gastrectomy for SMT were planned.

**Figure 2:**
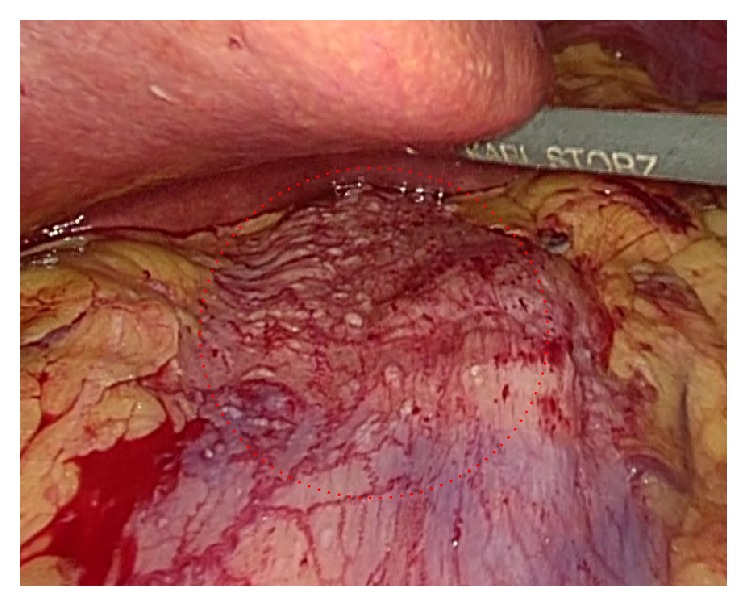
Intraoperative picture. There were multiple small nodules on serosal surface of upper stomach.

During the operation, multiple small nodules were identified on the serosal surface of the upper stomach (Figure 2). Most nodules were resected by proximal gastrectomy. There were no apparent abnormalities on the serosal surface of the small intestine or colorectum.

**Figure 3:**
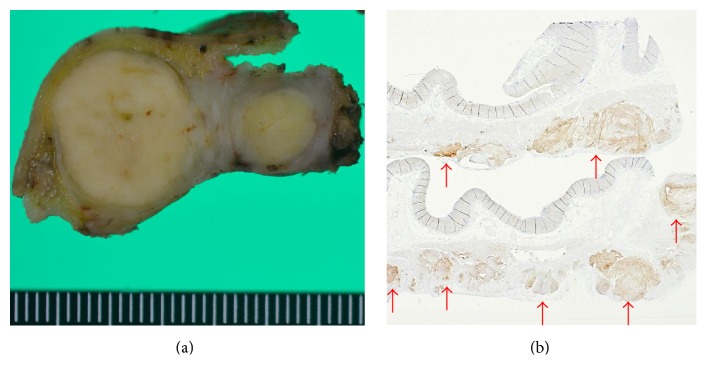
(a) SMT of the middle gastric wall contained two intramural lesions (1.4 × 1.2 cm and 0.8 × 0.6 cm). There were no findings indicating tumor rupture in these two lesions. (b) Multiple nodules located in subserosa of upper stomach. These nodules were positive for KIT (CD117).

**Figure 4:**
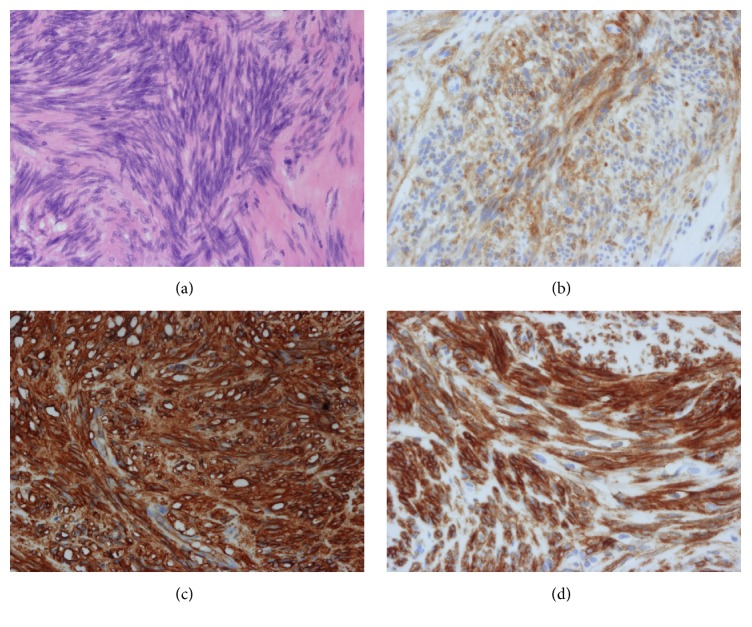
Histopathological analysis. (a) The tumors consisted of spindle cells. (b) Positive for KIT (CD117). (c) Positive for CD34. (d) Positive for DOG-1.

The result of a histopathological examination of the upper gastric lesion was consistent with NET G1; the MIB-1 index was 2%, without any lymph node metastases. In contrast, SMT of the middle gastric wall contained two intramural lesions (1.4 × 1.2 cm and 0.8 × 0.6 cm). These SMTs were compatible with GISTs; three mitotic figures in 50 HPF were seen. There were no findings indicating tumor rupture in these two lesions (Figure 3(a)). There were 90 or more small nodules on the gastric serosal surface, which were diagnosed as GISTs. These consisted of spindle cells positive for KIT (CD117), CD34, and DOG-1 (Figures 3(b) and 4). All these small nodules located under the serosa confirm that they were not a peritoneal metastasis.

Analyses of the c-*kit* gene and* platelet-derived growth factor receptor α* (*PDGFRA*) gene were performed in one GIST. There were no alterations in either the c-*kit* gene (exons 8, 9, 11, 13, and 17) or* PDGFRA* gene (exons 12, 14, and 18). The patient's normal gastric mucosal tissue also showed no mutations in the c-*kit* gene (exons 8, 9, 11, 13, and 17) or* PDGFRA* gene (exons 12, 14, and 18), confirming that this was not a case of familial GISTs with a germline mutation in the c-*kit* or* PDGFRA* gene.

The patient is being followed up without the administration of a tyrosine kinase inhibitor.

## 3. Discussion

GIST is the most common mesenchymal tumor in the digestive tract, originating from the interstitial cell of Cajal [4]. Sporadic GISTs are most commonly located in the stomach (60–70% of cases), followed by the small intestine (20–25%) and other locations [5]. In sporadic GISTs, 85–90% of cases have mutations in the c*-kit* gene. In addition, 35–62.5% of cases without c*-kit* gene mutations have mutations in the* PDGFRA* gene [6].

On the other hand, GISTs in NF 1 patients differ from sporadic GISTs in several aspects. A PubMed search of the literature revealed 126 case reports concerning GISTs associated with NF 1 (keywords: “Gastrointestinal stromal tumor” and “Neurofibromatosis 1”; language: English). Only 11 (8.7%) patients had gastric GISTs (Table 1) [1, 3, 7–12], of whom seven had multiple GISTs in the stomach and four had only one gastric GIST. Six patients also had GISTs in the small intestine. One patient had GISTs on another site, but with no description of the site involved. In contrast, 120 (95.2%) patients had GISTs in the small intestine, including the duodenum. In addition, there were two or more GISTs in 82 (65.1%) patients. Mutations in the c*-kit* gene were detected in only 2 of 51 patients (3.9%), and those in the* PDGFRA* gene were not detected (0/47).Thus, typical GISTs associated with NF 1 are located in the small intestine, show multiplicity, and have a mutation in neither the c*-kit* nor* PDGFRA* gene. Our NF 1 case with more than 90 GISTs on the serosal surface of the stomach is extremely unusual. To the best of our knowledge, there are no similar case reports.

GISTs associated with NF 1 are generally of low grade [3]. Our NF 1 case was also of low grade, with a maximum GIST size of 1.4 cm and with 3/50 HPF mitotic figures. In addition, GISTs without a mutation in the* c-kit/PDGFRA* gene appear to respond less well to a tyrosine kinase inhibitor than GISTs with this mutation. Therefore, a tyrosine kinase inhibitor may show no effect on GISTs associated with NF 1 [13]. In our case, left lesions or recurrences on the residual stomach were the major concerns. Nevertheless, we did not perform adjuvant chemotherapy with a tyrosine kinase inhibitor because the resected GISTs were low risk and a response to a tyrosine kinase inhibitor could not be guaranteed. For GISTs with NF 1, the importance of a routine follow-up is unknown. Our plan of follow-up for our patient is CT every 6 months for 5 years. When recurrences are detected, we will observe them unless they cause some symptoms such as obstruction or bleeding because multiple and metachronal recurrences are anticipated.

Familial and multiple GISTs caused by germline mutations in the c*-kit* or* PDGFRA* gene have been reported [14]. In such situations, multiple GISTs develop in both the stomach and the small intestine. All multiple GISTs have the same mutation in the c*-kit* or* PDGFRA* gene. Moreover, patients display the same mutation, even in the normal tissue. In the current case report, there was no mutation in the c-*kit* or* PDGFRA* gene not only in the GIST tissue but also in the normal gastric mucosa. This indicates that this is not a case of familial GISTs caused by germline mutations in the c*-kit* or PDGFRA gene.

In summary, we encountered an extremely rare case of multiple gastric GISTs associated with NF 1.

## Figures and Tables

**Table 1:** Cases of gastric GISTs in patients with NF 1.

Case number	Age	Sex	Total number of GISTs	Site	Number of GISTs	Size (cm)	Genetic analysis		Reference number
							*c-kit*	*PDGFRA*	
1	32	F	4	Stomach	ND	2–10	NE	NE	[7]
				Jejunum	ND				

2	82	F	Numerous	Stomach	ND	0.5–2.5	WT	WT	[7]
				Small intestine	ND				
				Colon	ND				

3	77	M	5	Stomach	ND	0.3–2.0	WT	WT	[7]
				Esophagus	ND				
				Jejunum	ND				
				Ileum	ND				

4	64	M	>100	Stomach	ND	0.1–3.5	WT	WT	[7]
				Small intestine	ND				
				Colon	ND				

5	58	M	5	Stomach	2	0.3, 3.0	WT	WT	[8]
				ND	3	ND			

6	64	F	Multiple	Stomach	1	11	WT	WT	[9]
				Small intestine	Multiple	Maximum 3.5			

7	40	F	1	Stomach	1	2.5	WT	WT	[10]

8	67	M	1	Stomach	1	Voluminous	NE	NE	[11]

9	71	M	1	Stomach	1	3.6	NE	NE	[3]

10	38	F	1	Stomach	1	8	NE	NE	[12]

11	59	F	4	Stomach	2	0.2, 0.5	WT	WT	[1]
				Jejunum	2	0.8, 3.0			

ND: not described, NE: not examined, and WT: wild type.
